# Forms of Continued Fever, and Their Definitions

**Published:** 1868-05

**Authors:** James Stark


### Forms of Continued Fever, and their Definitions.

BY JAMES STARK, M. D., F. R. S. E.

(Edinburgh Medical Journal, July, 1867.)

From the want of good definitions of the various forms of continued fever, many medical practitioners find themselves at a loss to distinguish one from another, and so confound all together. It is, however, very desirable that every one should be able to recognize all the several forms, as each requires its own special treatment. Despairing of the Royal College of Physicians of London completing the work, in which it has for years been engaged, and knowing how unwilling a public body is to commit itself in print, Dr. Stark has, on his own responsibility, drawn up the following summary of the forms of continued fever, and has appended definitions in such terms as have always enabled him to recognize them in his own practice. The distinctive names have been chosen as unlike each other as possible, inasmuch as great confusion occurs when the titles of the respective forms only differ in their terminal syllables, as is the case when the term typhus, typhia and typhinia are used.

First.—*Typhus Fever* (also called spotted, maculated, low, putrid, brain, and nervous fever.)—A continued fever, characterized by great prostration; with a mulberry or measly rash appearing early on the skin, *but not in successive crops*, and generally remaining visible during the course of the disease. Bowels generally confined, and no lesions therein found on dissection.

Second.—*Enteric Fever* (also called typhoid, typhia, gastro-enteric and gastric fever, abdominal typhus, and dothinenteritis.) A continued fever, characterized by rose-colored spots appearing chiefly on the trunk of the body about the end of the first week, *and in succsssive crops*, each crop disappearing in three or four days. Bowels often loose, and stools light colored. Enlargement and ulceration of the aggregate glands of the ileum usually met with on dissection.

Third.—*Relapsing Fever* (also called short, or relapsing typhus, bilious typhoid fever, typhinia, and synocha.)—A continued fever of short but varying duration, characterized in general by an absence of eruption, but by an intermission of all the symptoms, and an apparent temporary return to health for a longer or shorter period; by the fever relapsing after five to eight or more days, and by more than one relapse occurring before health is restored. Skin often dark, or more or less yellow. As occurs in intermittents, there is a strong tendency to enlargement of the liver and spleen.

Fourth.—*Gastric Fever* (called also bilious fever.)—A continued fever attended by gastric and biliary derangement, with no eruption, and no tendency to relapses. Skin often more or less yellow.

Fifth.—*Simple Continued Fever* (also called simple typhus, and simple fever.)—A continued fever, without eruption, and without a tendency to relapse.

Sixth.—*Febricula.* A simple fever, without eruption, of from one to, at the most, three or four days' continuance.

Seventh.—*Infantile Fever* (also called infantile remittent fever.) Fevers occurring in childhood, characterized by daily exacerbations and remissions, with a tendency to diarrhœa. Cases of enteritis are often mistaken for this form.

Though these are the forms which prevail in this country, there is an eighth form, which is common on the continent of Europe, and which is the most fatal of all, usually cutting off 40 per cent. of those it attacks, and proving fatal on the third or fourth day of the attack. This is the epidemic brain fever or cerebro-spinal meningitis, so well known in Russia, Norway, Sweden, etc., and which constitutes no small portion of every epidemic of fever which attacks these countries. Occasional cases occur in this country; but we still want a good definition of the disease.—*Half-Yearly Abstract.*

